# Osteoblast-like Cell Response to Titania-Ca-Modified Siloxane Biohybrids

**DOI:** 10.3390/life16101733

**Published:** 2026-10-15

**Authors:** Ricardo Iván Gutiérrez-Ventura, Hugo Iván Arrieta-Oliva, Daniela Anahí Sánchez-Téllez, Manuel Alejandro Mosso-Pani, Blanca Estela García-Pérez, Lucía Téllez-Jurado

**Affiliations:** 1 Departamento de Ingeniería en Metalurgia y Materiales, Escuela Superior de Ingeniería Química e Industrias Extractivas, Instituto Politécnico Nacional. Av. Instituto Politécnico Nacional S/N, Lindavista, Mexico City 07738, Mexico; rgutierrezv@gmail.com (R.I.G.-V.); danielatellez06@gmail.com (D.A.S.-T.); 2 Departamento de Microbiología, Escuela Nacional de Ciencias Biológicas, Instituto Politécnico Nacional. Prolongación de Carpio y Plan de Ayala S/N, Casco de Santo Tomás, Mexico City 11340, Mexico; hugo.oliva67@gmail.com; 3 Laboratory of Genomics, ODIN Bioscience, Miami, FL 33166, USA; qbp.mosso@hotmail.com

**Keywords:** siloxane hybrids, titania, osteoblasts, tissue regeneration, tissue engineering

## Abstract

This study examined the response of osteoblast-like cells to siloxane-based biohybrid materials obtained via the sol–gel process and modified with titania or with titania and calcium. The biohybrids’ ability to form apatite and their degradation process were assessed in vitro via immersion in PBS and SBF. Characterization was performed using XRD, FT-IR, and SEM. The Siloxane-Ti biohybrid exhibited rutile crystalline phase formation, high stability, and resistance to hydrolytic degradation, but did not induce apatite growth. In contrast, the Ca-containing biohybrid showed a vitreous structure with significant surface morphology changes and increased degradability. The Ca-modification enhanced the precipitation of apatite layers, which was attributed to the presence of Ti-OH and Si-OH groups. Immersion of the Siloxane-Ti-Ca biohybrids in PBS produced a flake-like apatite-like mineral structure from the first day of immersion, while immersion in SBF led to a cauliflower-like apatite structure after 28 days. Osteoblast response was evaluated through cell adhesion, proliferation, and differentiation on the hybrid materials. The Siloxane-Ti hybrid promoted osteoblast adhesion and proliferation, whereas the Siloxane-Ti-Ca reduced cell viability and osteonectin expression at 21 days. The Siloxane-Ti hybrid also increased osteonectin expression at early stages, with osteopontin and osteocalcin expression emerging at later stages, indicating progression from early to mature stages stimulated by the hybrid material. The findings suggest that titania enhances the biocompatibility of siloxane materials and shows significant potential for bone tissue regeneration without added growth factors.

## 1. Introduction

Bone tissue engineering is an evolving field that requires the development of innovative alternative biomaterials with enhanced biocompatibility and bioactivity [1]. Hybrid materials, formed by combining organic and inorganic components, meet these requirements due to their molecular-level homogeneity, adaptive structure, simple synthesis, and favorable biological properties applicable to bone regeneration [2,3,4].

Among these organic–inorganic systems, hybrid materials based on siloxane systems (TEOS [Tetraethyl orthosilicate]-PDMS [Polydimethylsiloxane]), also known as Organically Modified Silicates (ORMOSILs), show outstanding mechanical and bioactive properties related to both the abundant hydroxyl (–OH) groups bonded to the silica networks (silanol, Si-OH groups) and the negatively charged surfaces, surpassing those of many bioceramics [5,6]. ORMOSILs typically exhibit a flexible degradation rate when their hydrophilic/hydrophobic character is modified by selecting components and adjusting the organic/inorganic ratios [7].

We previously demonstrated two behaviors: the incorporation of zirconia into ORMOSIL hybrids enhanced cell adhesion and growth of in vitro osteoblast cultures, and 2) adding calcium into the ORMOSIL hybrids promoted the development of an apatite-like structure, decreasing cell growth and osteonectin expression [3].

The incorporation of titania (TiO_2_) and CaO into siloxane networks via sol–gel processing must improve both the mechanical resilience and the surface bioactivity of these hybrids [8,9]. Titania enhances the formation of hydroxylated surfaces that act as nucleation sites for apatite, which improves the condensation of siloxane units, increasing mechanical strength and promoting structural stability, known as an osteoconductive material [10]. Calcium incorporation increases hydrophilicity and introduces Ca^2+^ ions that interact with physiological fluids and proteins, accelerating mineral deposition, enhancing surface reactivity, and promoting local supersaturation with respect to bone-like apatite phases [9,10,11]. Importantly, it has been demonstrated that the presence of either titania or calcium alone can provide apatite-inducing capacity [12]. The nature of the calcium precursors influences the final structure of the siloxane matrix. Calcium nitrate requires heat treatment up to 400 °C to eliminate residual nitrates, increasing Q4 units and decreasing D-Q bonds, which are characteristic of the siloxane network, while titanium enhances thermal stability and maintains the integrity of the network [13].

Several studies have examined sol–gel-derived siloxane hybrids containing TiO_2_ and/or CaO, reporting structural modifications, apatite-inducing capacity in simulated body fluid (SBF) [9,14], and, in a few cases, interactions with osteoblast-like cells [4]. Almeida et al. [4], for example, demonstrated that cell adhesion and proliferation are possible even on hydrophobic siloxane–titania–calcium hybrids, attributing this to hydrophilic calcium-rich silica domains at the nanoscale that trap water, where the proteins adhere, starting the interaction between cells and the material surface. The study uses organic salt as a calcium precursor to avoid heat treatment to eliminate nitrate residues, and no biological test was carried out on a hybrid containing only titanium.

Additionally, other researchers have demonstrated that the anatase crystalline phase of titania enhances apatite induction [9,10,11], whereas the rutile phase, formed at higher temperatures during the process, displays lower bioactivity due to reduced Ti–OH group density [14]. It is known that anatase generates reactive oxygen species (ROS), inducing oxidative stress and toxicity. In contrast, the rutile phase is less reactive and more stable, inducing less oxidative stress [15,16]. Moreover, the amorphous titania phase produces minimal radicals under UV light, resulting in low or negligible cellular toxicity, and is considered a safer choice for topical applications [17]. Furthermore, favorable biological responses have been demonstrated in siloxane hybrids with a similar composition to the present work [3]. Nevertheless, most prior studies have focused either on physicochemical aspects or apatite precipitation in SBF, with relatively few researchers providing a direct correlation between structural modifications, apatite formation in different media, and osteoblast responses.

In this work, we address this knowledge gap by synthesizing biohybrid-based siloxane via the sol–gel method, using nitrate salt as a calcium precursor, modified with either titania (Siloxane-Ti) or titania plus calcium (Siloxane-Ti-Ca), dried at controlled temperatures using a heating ramp of 40, 80, and 110 °C, and without heat treatment at higher temperatures. We systematically evaluate their structural characteristics, in vitro degradability in PBS, ability to form calcium apatite in SBF, and their interactions with human osteoblasts, including adhesion, proliferation, and differentiation without the presence of growth factors. Our study contributes to the knowledge on the use of TiO_2_ and Ca^+2^ incorporation into biohybrid siloxanes and suggests their potential usage for bone tissue regeneration.

## 2. Materials and Methods

### 2.1. Synthesis of Hybrids

Siloxane hybrid samples were synthesized by the sol–gel method using tetraethyl orthosilicate (TEOS, Sigma-Aldrich, USA) and titanium isopropoxide (Ti(OiPr)_4_, Sigma-Aldrich, USA) as inorganic precursors, and hydroxy-terminated polydimethylsiloxane (PDMS, Mn = 550 g/mol, Sigma-Aldrich, USA) as the organic component. Hydrochloric acid (HCl, Merck, Germany) served as the catalyst, 2-propanol (iPrOH, Sigma-Aldrich, USA) as the solvent, and distilled water as the hydrolyzing agent. For calcium incorporation, calcium nitrate tetrahydrate (Ca(NO_3_)_2_·4H_2_O, Sigma-Aldrich, USA) was added. The mass ratio of inorganic/organic in Siloxane-Ti was fixed at (0.45 TEOS + 0.20 Ti(OiPr)_4_)/0.35 PDMS, while Ca^2+^ addition corresponded to 5 wt% of the precursor mixture, resulting in ~1 wt% Ca in the final siloxane-Ti-Ca hybrids. In addition, the Ti/Ca ratio was 3.5, and the molar ratios of inorganic precursors/HCl/(iPrOH)/H_2_O were 1/0.3/4.5/3 (Table 1). The calculated amounts (g/g) to prepare the siloxane-Ti hybrid were [20.5 g TEOS, 9.2 g Ti(OiPr)_4_, and 5.6 g PDMS]. For the Siloxane-Ti-Ca hybrid, the same quantities of the reagents mentioned above were used, but with the addition of 2.2 g of Ca(NO_3_)_2_·4H_2_O.

Conversely, control samples (Siloxane and Siloxane-Ca) for cell assays are already reported in previous work [3]. The samples were prepared in the same way explained above, using the same mass ratio of inorganic/organic (0.65 TEOS)/0.35 PDMS) for the siloxane control and with the addition of ~1 wt% Ca for the siloxane-Ca control.

The synthesis procedure involved the preparation of three initial solutions: (i) PDMS + TEOS + iPrOH, (ii) HCl + H_2_O + iPrOH, and (iii) Ti(OiPr)_4_ + iPrOH, each homogenized by magnetic stirring for 15 min at room temperature. For calcium-containing hybrids, a fourth solution of Ca(NO_3_)_2_·4H_2_O + H_2_O + iPrOH was prepared. The PDMS–TEOS solution was first placed in a thermostatic bath at 80 °C under mechanical stirring (600 rpm). After that, the other solutions were added dropwise in alternating sequences over 30 min. The mixture was stirred for 10 additional min under the same conditions and then poured into polypropylene containers to begin gelation. Gels were aged for 7 days at room temperature, followed by preliminary drying at room temperature (8 days) and finally staged drying at 40 °C for 3 days, 80 °C for 5 days, and 110 °C for 3 days. The resulting samples were named Siloxane-Ti (Ti-modified) and Siloxane-Ti-Ca (Ti + Ca-modified). The characterization of the hybrids was performed before and after the immersion experiments using scanning electron microscopy coupled with an energy-dispersive spectrometer (SEM-EDS, JEOL JSM-6701F and JEOL JSM-6300, Japan Electron Optics Laboratory Co., Ltd., Japan), Fourier transform infrared spectroscopy (FT-IR/ATR, PerkinElmer Frontier, PerkinElmer, Waltham, MA, USA), and X-ray diffraction (XRD, Bruker D8 Focus, Bruker AXS, Karlsruhe, Germany) (Figure 1).

### 2.2. Stability and Mineralization Evaluation

The stability (physical integrity) and mineralization ability of Siloxane-Ti and Siloxane-Ti-Ca hybrids were assessed by immersion in Kokubo’s simulated body fluid (SBF) [18] and phosphate-buffered saline (PBS) under physiological conditions (36.5 ± 0.5 °C, pH 7.4) for up to 28 days [19]. The materials (0.5 cm^3^, SA: V of 12:1) were placed in amber glass jars with screw tops; then, 30 mL of each solution (PBS or SBF) was added to each glass jar, and the jars were sealed and placed in a humidity-controlled incubator at 36.5 ± 0.5 °C. Once the incubation period finished, the material was removed from the glass jar and subjected to a drying process at 110 °C for 24 h, and then the samples were weighed. No medium renewal or rinsing was performed.

Variations in pH and weight loss were recorded at 1, 14, and 28 days. Weight loss (WL, %) was calculated as:

%WL = (W1 − W2)/W1 (100)

where W_1_ is the initial weight, and W_2_ is the weight after drying. Triplicate samples were analyzed for each condition. Post-immersion surfaces were examined by Scanning Electron Microscopy (SEM-EDS) (JEOL JSM-6701F), X-Ray diffraction (XRD) (Bruker D8 Focus), and Fourier Transform Infrared (FT-IR/ATR) (PerkinElmer Frontier).

### 2.3. Osteoblast Culture Conditions

Human osteoblast-like osteosarcoma cell line (MG-63, CRL-1427 ATCC, Manassas, VA, USA) was cultured in α-MEM (Hyclone, Cytiva, UT, USA) supplemented with 10% fetal bovine serum (FBS, Gibco Thermo Fisher Scientific, Waltham, MA, USA) at 37 °C, 5% CO_2_. Cells were detached using 0.05 g/L trypsin–EDTA and subcultured according to ATCC protocols. For all experiments, osteoblast-like cells were used between 5 and 15 passages.

### 2.4. Cell–Material Interaction

Monolith hybrids were fragmented into pieces of approximately 0.5 cm^3^ to evaluate their biocompatibility with osteoblast-like cells. Fragments were sterilized by immersion in 96% ethanol and exposure to UV radiation for 30 min. Then, the sterilized fragments were seeded in 96-well plates and incubated overnight with 200 µL of α-MEM supplemented with 10% FBS at 37 °C, 5% CO_2_ to eliminate the trapped remnants from the synthesis process. Afterwards, the medium was removed, and osteoblast-like cells (5 × 10^4^ cells/mL) were seeded on the materials in 96-well plates and maintained for 7, 14, and 21 days with daily medium changes. Fragments of siloxane hybrids without TiO_2_ and Ca^+2^ were used as controls. These fragments were incubated under the same conditions as those incubated with cells. Experiments were conducted in triplicate.

### 2.5. Assessment of Cell Viability

To assess cell viability, osteoblast-like cells grown on materials after 7, 14, and 21 days were stained with two different nucleic acid dyes: 5 μM SYTO9 (Invitrogen, Carlsbad, CA, USA) and 30 μM propidium iodide (Sigma-Aldrich, St. Louis, MO, USA). Samples were prepared for further analysis by confocal microscopy (LSM5, Pascal, Zeiss, Germany) as previously described [3]. Additionally, 100 cells were quantified in three fields to calculate the percentage of viable and dead cells present on the siloxane hybrid materials surface.

### 2.6. Proliferation Assays

The cell proliferation supported by hybrid materials was analyzed by the 3-(4,5-dimethyl-2-thiazolyl)-2,5-diphenyl-tetrazolium solution (MTT; 5 mg/mL, Sigma-Aldrich, St. Louis, MO, USA) method. Osteoblast cells were cultured at a density of 5 × 10^4^ cells/mL per hybrid material and incubated at 37 °C and 5% CO_2_ for 24 h. After incubation, materials were transferred to a new plate to allow the proliferation of only those cells that initially adhered to the materials, and this time was considered day 1. Then, cells were incubated for 7, 14, and 21 days at 37 °C and 5% CO_2_. After each incubation time, 100 µL of MTT was added, and samples were incubated again for 3 h at 37 °C. Subsequently, 100 µL of dimethyl sulfoxide (DMSO) was added to dissolve the formazan precipitates formed. Absorbance was determined in an ELISA plate reader (Multiskan Spectrophotometer, Thermo Fisher Scientific, Waltham, MA, USA) at 590 nm. The proliferation rate was calculated to estimate the increase in the number of osteoblast-like cells grown on the hybrid surfaces. Optical density values from days 7, 14, and 21 were normalized to the optical density value obtained on day 1, which represented the cells initially adhered to the hybrids.

### 2.7. Cytoskeletal Organization and Cell Morphology

Cytoskeletal organization was evaluated through actin filament staining with 10 ng/μL phalloidin–TRITC (Sigma Aldrich, St. Louis, MO, USA) for 20 min, nuclei with DAPI (Vector laboratories, Burlingame, CA, USA), and visualized by confocal microscopy. Osteoblast morphology and surface attachment were further examined by SEM (JEOL 5800LV, GenTech Scientific, NY, USA) as we previously reported [3].

### 2.8. Osteogenic Marker Expression

Cell differentiation was evaluated by the expression of osteonectin (SPARC), osteopontin (OPN), and osteocalcin (OCN) at 1, 7, 14, and 21 days [4]. Osteonectin expression was detected by immunofluorescence using a primary anti-SPARC antibody (H-90, sc-25574, Santa Cruz Biotechnology, Inc., CA, USA) at a 1:100 dilution. Then, a TRITC-conjugated secondary antibody (SC-2367, Santa Cruz Biotechnology, Dallas, TX, USA) was used at a 1:200 dilution. Afterwards, the cells were incubated with SYTO9 (5 μM, Invitrogen, Carlsbad, CA, USA) to stain the cell nuclei. Samples were analyzed under a confocal system coupled to inverted microscopy (LSM5, Pascal, Zeiss, Germany).

OPN and OCN secretion in supernatants was determined by a Dot blot assay. The nitrocellulose membranes were sensitized with 50 μL of total protein obtained from three wells of the same condition. Then, sensitized membranes were overnight probed with 1:250 dilution of anti-OPN (OPN AKm2A1, Santa Cruz Biotechnology Inc. CA, USA) or 1:250 dilution of anti-OCN (OCN FL-100, Santa Cruz Biotechnology Inc. CA, USA). Finally, the protein expression was revealed using a goat anti-mouse coupled with HRP at a 1:2000 dilution or a goat anti-rabbit coupled with HRP at a 1:1000 dilution, respectively, for 2 h at 37 °C. Signal development was performed using HRP substrate (Bio-Rad Laboratories, Hercules, CA, EE. UU.) and imaged with a ChemiDoc system (Bio-Rad Laboratories, Hercules, CA, EE. UU.). Osteoblasts cultured in the maintenance medium served as differentiation controls. The pixel intensity of the dots revealed on the membranes was semi-quantified by digital densitometry using ImageJ 1.52a software.

## 3. Results and Discussion

### 3.1. Structural Features of Titania–Siloxane Hybrids

Previous studies on siloxane hybrids modified with P_2_O_5_ or TiO_2_ have shown that adding ~5 wt% CaO is sufficient to confer apatite-forming ability upon immersion in physiological-like media [20,21]. Tamayo et al. [12] reported up to 15 wt% CaO in siloxane hybrids with TiO_2_, also reported that 5 wt% possesses the ability to form apatite and demonstrated that CaO- and TiO_2_-free siloxanes are non-bioactive. Moreover, CaO and TiO_2_ enhance surface basicity and promote bioactivity, as well as maintain the ionic content within an optimal range. Excess Ca(NO_3_)_2_, used as a CaO precursor, caused undesirable salt precipitation with increasing CaO content. Arrieta et al. [3] reported the use of 1 wt% Ca in similar hybrid materials with osteoconductive response in the presence of osteoblastic cells. Furthermore, the use of 1 wt% Ca allows better control over degradation, thereby maintaining the hybrid’s integrity and minimizing residual nitrates from the precursor salt, facilitating their removal via washing prior to cell seeding without requiring high-temperature heat treatments (400 °C) [13]. Additionally, the lower content of precursor salt helps preserve the buffering capacity of the PBS and keeps pH values within optimal ranges. This occurs because Ca ions interact with phosphates in the solution while nitrates remain dissolved, forming potassium and sodium nitrates (ionic species already present in the PBS) without causing abrupt pH fluctuations [22].

On the other hand, the intermediate TiO_2_ contents created favorable acid–base conditions for apatite nucleation. Based on these findings, our choice of TiO_2_ incorporation was guided by enhanced surface reactivity and by its reported biocompatibility, a positive soft-tissue response, and potential for osseointegration [23].

X-ray diffraction (XRD) patterns in Figure 2 revealed significant differences between the siloxane hybrid bonded to titania and the siloxane hybrid-titania plus calcium. The Siloxane-Ti hybrid shows broad peaks at 2θ ≈ 27°, 36°, 41°, 44°, 54°, 56°, 63°, and 69°, due to the incipient rutile crystalline phase formed (PDF card 21-1276) within an amorphous siloxane matrix. The presence of the rutile phase of TiO_2_ is unusual for sol–gel systems processed at low temperatures [24], and it may be related to the multi-stage drying program used during the dry stage of the sample synthesis (see Section 2.1). Similar low-temperature crystallization phenomena have been observed for titania-containing gels treated in hot water conditions, resulting in the formation of anatase and rutile phases [24,25]. Rutile crystallizes at low temperatures (40–80 °C) when titanium alkoxides (tetrabutoxide or isopropoxide) are used in a water bath, with acidity and hydrolysis kinetics controlled during the sol-gel process [24]. The water bath ensures uniform heat transfer and slows solvent evaporation and Ti-O-Ti bond condensation, allowing the chains to arrange themselves into the thermodynamically stable rutile phase, given that the acidic medium predisposes the titanium octahedra toward the rutile structure [26]. Additionally, using a strong catalyst such as HCl promotes hydrolysis of the titanium alkoxide and the formation of edge-sharing octahedral complexes, thereby favoring rutile over anatase structural packing [25].

In contrast, Siloxane-Ti-Ca remained predominantly amorphous, resembling vitreous silica [7,27], with no evidence of the rutile crystalline phase, indicating that calcium acted as an interfacial modifier, modulating phase separation in the polymer network [28] and preventing titania crystallization (Figure 2).

During the polycondensation reactions, the incorporation of calcium precursor salt influences condensation kinetics, acting as a modifier/modulator of condensation kinetics, affecting the forming hybrid network and promoting the dispersion of organic and inorganic components [28]. After that, during the drying process, calcium ions remain in local domains, weakly coordinated with hydrophilic silanol (Si-OH) groups, which modifies the electrostatic forces and modulates phase separation between hydrophobic forces and hydrophilic components (Si-OH), resulting in a macroscopically uniform hybrid structure [28,29,30]. The synthesis process generates clusters dispersed within the matrix, altering macroscopic properties through the control of phase dispersion [30]. Residual nitrates remain freely trapped and may recrystallize during the drying stage. At 110 °C, crosslinking of the siloxane network via residual silanol groups is favored, and the dehydrated salt is deposited in amorphous domains [31,32].

### 3.2. Vibrational Signatures and Network Integrity

The analysis of Fourier-transform infrared spectroscopy (FT-IR) confirmed that the siloxane system has a hybrid nature. Siloxane-Ti spectrum displayed Si–O–Si vibrations (1180 cm^−1^, 1014 cm^−1^), Si–C bands from PDMS (1260 cm^−1^, 793 cm^−1^), and Ti–O modes (400–650 cm^−1^). Additionally, Siloxane-Ti-Ca exhibited nitrate-related peaks at 1424 and 1340 cm^−1^ from the calcium precursor due to the asymmetric stretching of nitrate, ν_3_ mode. Also, broad bands at 3300 cm^−1^ due to symmetric and asymmetric stretching of H-O-H, ʋ_1_ and ʋ_3_ modes, and 1640 cm^−1^ of the bending vibration of H-O-H, δ(H_2_O), were observed. Also, the band at 1640 cm^−1^ may be related to the bending mode of ʋ_2_ of the H-O-H bonds of the structural water bound to the calcium in the hydrated salt. This salt dissolves upon incorporation into the matrix during the synthesis, but in the drying process, the salt re-precipitates and tends to hydrate upon contact with the wet gels [33,34]. A shoulder between 1102–1080 cm^−1^ is due to a superposition of the asymmetric stretching of Si-O-Si bonds and the deformation vibrations of the hydroxyl groups (Ti-OH) from Ti-O-H groups [35,36]. This indicates that these groups may serve as potential nucleation sites for mineralization [36,37,38]. Moreover, the stretching vibration of the Si-O bond in the Si-O-Si and Si-O-C bonds was observed at 1014 cm^−1^. The band attributed to the copolymerization of D-Q units (D unit from PDMS and Q unit from TEOS) was observed at 850 cm^−1^, confirming the linkage of PDMS-Si-O-Si-TEOS due to the siloxane hybrid structure formed. Finally, the bands located at 400 cm^−1^ and 650 cm^−1^ correspond to the Ti-O vibrational mode (Figure 2B).

On the other hand, SEM analysis revealed that the Siloxane-Ti exhibited a smooth and dense morphology with small silica-derived particles, whereas the Siloxane-Ti-Ca showed a porous structure with interconnected particles and agglomerate domains (Figure 2C). The presence of both Ti and Ti-Ca, respectively, is observed in the elemental mapping in Figure 2D, and they are distributed homogeneously throughout the siloxane network. As can be seen, the calcium addition promotes a less interconnected siloxane matrix compared to that produced solely with the presence of titania [12]. Moreover, it has been reported that the presence of titania in siloxane networks from metal alkoxides acts as a network crosslinker by means of hydrolysis and condensation reactions, promoting a denser and less porous polymer matrix [39].

### 3.3. Hydrolytic Stability and Medium Interaction

To assess the hydrolytic degradation and chemical stability of siloxane hybrid materials, the weight changes after immersion in PBS (Phosphate-Buffered Saline) [20] were monitored for different periods (1, 7, 14, 21, and 28 days) (Figure 3). The Siloxane-Ti hybrid showed minor changes in both weight (Figure 3A) and pH, as the latter remained between 7.25 and 7.4 (Figure 3B). This hybrid exhibits a slight weight gain on days 1, 5, and 28 (~2%, 5%, and ~1%, respectively). Nevertheless, this could also be attributed to interactions between Si-OH and Ti-OH groups, located on the material surface, and phosphate ions, as well as hydrogen bonds with PBS-water, remaining even after drying due to the presence of hydrophobic PDMS, limiting their diffusion. Also, this slight weight gain may be related to the incorporation of dissolved salts (K or Na) from PBS. However, this almost zero weight loss of the Siloxane-Ti is attributed to its dense structure, conferring chemical stability, and could be related to the titanium alkoxide activity as a crosslinking agent of the siloxane chains during condensation reactions in the sol-gel synthesis of the hybrid [13]; but, as mentioned above, it can also be attributed to the relationship between the hydrophobic/hydrophilic character of the composition [40].

In contrast, the presence of Ca ions led to a weight loss of approximately 15% upon immersion in PBS after 28 days. In the first 3 days, the residual nitrates from the precursor salt were dissolved, as observed in FT-IR analysis (Figure 2B). On day 5, the weight gain is related to the calcium-phosphate/apatite-like deposits. After that time, the weight remains almost constant until day 21. Finally, on day 28, a loss of weight was observed. This could be related to a combination of material degradation and mineral deposits.

This weight loss falls within the ranges reported for systems of similar composition after assaying in SBF [21], attributed to degradation kinetics governed by two stages: (1) leaching of the calcium precursor salt and (2) gradual hydrolysis of the silicates derived from TEOS. Meanwhile, the PDMS regulates water diffusion due to its hydrophobic nature, preventing structural collapse and stabilizing weight loss at later stages of the assay. The reported weight loss range is optimal, as it allows the material to maintain its physical integrity while the ion-exchange process for calcium phosphate nucleation takes place. A limitation of our work was that the content of Ca released to the solution was not determined. Thus, it is not possible to determine which of those processes is responsible for weight loss.

These findings align with a study on similar hybrid materials, which found that Ca-containing hybrid materials exhibit a significant decrease in their mechanical properties upon immersion in SBF compared to those without calcium [41]. This study attributed the changes in hybrid material properties to the activity of Ca^+2^ ions that act as structural modifiers and are then released from the network structure, promoting ion exchange with H_3_O^+^ and allowing water to infiltrate the network, resulting in the breaking of Si-O-Si bonds and thus material degradation.

As mentioned in the Section 2.1, the hybrid siloxane materials undergo drying at 110 °C to remove residual components. After this stage, no macroscale phase separation was observed; instead, a homogeneous dispersion of calcium ions was observed (EDS analysis in Figure 2D). According to the literature [28,29,30], during drying, the Ca^+2^ ions are available in porous spaces, physically adsorbed on the surface, onto silanol and titanol (Si-OH and Ti-OH) groups rather than acting as a network modifier.

Furthermore, during the interaction between siloxane hybrids and SBF (Simulated Body Fluid), they exhibit a similar behavior to that observed in the PBS assay. In Figure 3C, the Siloxane-Ti hybrid does not show noticeable weight loss; the minimal weight changes may be attributed to experimental error or minimal salt deposition (Na and/or K) between 3 and 7 days from the SBF or material degradation between 7 and 21 days. However, the Siloxane-Ti-Ca hybrid shows a greater weight loss (ranging from 12 to 20%) than that observed in the PBS assay. The initial weight loss (up to day 3) is due to the residual nitrates being released; until day 7, the weight loss could be related to material degradation, as the more open structure allows for a greater influx of solution into the cavities, alongside the greater ion exchange interaction between the Siloxane-Ti-Ca and the SBF components than with PBS, leading to the precipitation calcium-phosphate /apatite-like deposits until day 28, observed as a weight gain. The observed pH values throughout the test time (Figure 3D) show a slight increase on the first day. After that time, it remains almost constant. The minimal change in pH values, ranging from 7.4 to 7.6 in both siloxane hybrids, indicates interaction between the components of SBF and the materials [21].

### 3.4. Mineralization Response in PBS and Medium Interaction

The XRD patterns in Figure 4 track the structural evolution of the siloxane-hybrid materials during immersion in PBS and SBF. The Siloxane-Ti sample exhibited the crystalline phase of rutile (PDF card 21-1276) (Figure 2A), which persisted for the entire testing period in both media (Figure 4A,C); also, two new peaks were observed at 2θ in 32° and 46° due to primary reflections of halite (PDF card 05-0628) in the interaction between Siloxane-Ti and SBF on day 28 (Figure 4C). On the other hand, the Siloxane-Ti-Ca sample in PBS (Figure 4B) shows new broad and low- intensity diffraction peaks at 26°, 32°, and 46° in 2θ; the last two correspond to the overlap of primary reflections of halite (PDF card 05-0628) with those of calcium-phosphate/apatite-like deposits (PDF card 09-0432). This last phase is also observed in the interaction between the Siloxane-Ti-Ca and the SBF medium (Figure 4D) at 14 and 28 days.

FT-IR analysis of the Siloxane materials (Figure 4) showed minimal spectral changes over time, demonstrating the stability of their chemical bonds and resistance to hydrolytic degradation. However, in the interaction of the Siloxane-Ti-Ca material with both media (Figure 4B,D), the nitrate-related peaks at 1424 cm^−1^ and 1340 cm^−1^ from the calcium precursor disappeared on the first day of testing. Also, two new absorption bands appeared at 560 cm^−1^ and 600 cm^−1^. These bands are characteristic of the ʋ_4_ bending vibration in phosphate groups [42,43], providing evidence of interaction between PBS and SBF and the chemical changes associated with the incorporation of calcium-containing species in the siloxane matrix. These findings are associated with the bioactivity of materials in both media. PBS is used to assess the chemical stability of hybrids. Meanwhile, the material-SBF interaction makes it possible for the formation of calcium-phosphate/apatite-like deposits on the material’s surface, considering possible material bioactivity, which leads to a possible positive response for cell seeding. The phosphate (PO_4_^)−3^ groups in these precipitates show intense bands at 1000 cm^−1^ and 1100 cm^−1^ (asymmetric stretching of ʋ_3_ mode) and a shoulder at 962 cm^−1^ (symmetric flexion of ʋ_1_ mode) [42,43]. The siloxane network shows a broad and intense band between 1200 cm^−1^ and 900 cm^−1^ because of the overlapping of the bands at 1020 cm^−1^ and 1110 cm^−1^ due to the asymmetric stretching of Si-O-Si bonds. Therefore, slight changes in this broad band could be related to the calcium-phosphate/apatite-like deposits formed on the siloxane hybrid (see inserts in the FT-IR spectra, Figure 4) [12,20].

The lack of bioactivity in Ca-free hybrids reinforces the central role of calcium release in generating nucleation sites and driving mineralization, whereas rutile titania without accessible Ti–OH groups is insufficient for apatite induction.

SEM images before (Figure 2C) and after PBS immersion (Figure 5) show that the Siloxane-Ti material exhibited a dense, homogeneous, and smooth surface with small spherical silica particles formed during the syneresis and drying stages (Figure 2C). However, the dissolution of these superficial spherical silica particles was observed on day 1, which may have contributed to the slight pH variation in the PBS (Figure 3B). After this time, and until 28 days, no significant surface degradation was observed. On the other hand, the interaction between Siloxane-Ti and SBF shows some precipitates with irregular morphology (Figure 6) up to 28 days, attributed to sodium chloride (halite) according to XRD (Figure 4C) and EDS analysis (Figure 6B).

In contrast, the incorporation of calcium ions altered the hydrolysis-condensation reactions in the Siloxane-Ti-Ca material synthesis, increasing condensation rates and resulting in a less interconnected vitreous network. This led to an agglomerated particle morphology with high cavities (Figure 2C). Starting from the first day of immersion in PBS, a new phase with a platelet morphology, calcium-phosphate/apatite-like deposits [44], precipitated on the surface (Figure 5). These apatite-like deposits show incipient peaks corresponding to the principal reflections of apatite by XRD and the phosphate bands in FT-IR (Figure 4B) and continued to grow for 28 days until cauliflower-like morphologies formed (Figure 5B), demonstrating the calcium-phosphate/apatite-like deposits (bioactivity) of the Siloxane-Ti-Ca material. According to EDS analysis (Figure 5B), the Ca/P ratio after 1, 14, and 28 days in PBS was 1.55, 1.18, and 1.71, respectively. These values are in agreement with those of calcium phosphate reported in the literature [45].

Furthermore, from the first day up to 28 days of testing, the interaction between Siloxane-Ti-Ca and SBF exhibits incipient precipitates (Figure 6) with a tiny flake-like morphology [12,44], which are difficult to observe on the porous surface. These correspond to the presence of the calcium-phosphate/apatite-like, consistent with the XRD, FT-IR (Figure 4D), and EDS (Figure 6B) analyses. The EDS analysis (Figure 6B) shows that the Ca/P ratio of the Siloxane-Titania-Ca hybrid tested for 1, 14, and 28 days in SBF was 3.52, 1.51, and 1.52, respectively. The value observed on day 1 (3.52) falls outside the range of values for calcium phosphates [45]; these values may be related to the interaction of calcium in the hybrid material and the ions of SBF. Furthermore, the Ca/P ratio at 14 and 28 days suggests calcium-deficient hydroxyapatite [45].

As shown, apatite-like mineral precipitates are formed during the interaction of Siloxane-Ti-Ca in both media (PBS and SBF). The bioactivity of the Siloxane-Ti-Ca material in PBS can be explained by the release of Ca^2+^ ions from the hybrid network via ion exchange with H_3_O^+^ ions, due to a strong interaction between the calcium from the nitrate salt and the phosphate solution [9]. Unlike SBF, which emulates human blood plasma and includes multi-ionic components, PBS is a simpler solution of phosphates and chlorides that lacks calcium ions. Therefore, it does not provide free Ca ions to interact with the siloxane surface, which undergoes slight hydrolysis, forming some active Si-OH and Ti-OH groups on the surface, causing the release of the Ca^+2^ ions from the siloxane matrix into solution, attracted by the abundant phosphate in the medium. This process results in the direct chemical precipitation of calcium-phosphate/apatite-like through rapid proton exchange [21].

In the SBF, an ionic exchange process generates Si-OH and Ti-OH groups, which are known to induce apatite nucleation [9]. These groups enhance ionic interactions by forming a Si-OH-rich layer on the Siloxane-Ti-Ca surface. When these groups undergo deprotonation, they acquire a negative charge (Si-O-), which attracts calcium ions (Ca^2+^) from the solution by electrostatic interaction, forming a calcium-rich layer that captures PO_4_^−3^ or HPO_4_^−2^ ions from the SBF, saturating the medium and forming a mineralized apatite film on the siloxane-Ti-Ca surface [12,21].

The lack of bioactivity in the Ca-free material is consistent with studies on similar hybrid systems [41], which showed that only Ca-containing samples were able to induce apatite formation. The inability of the Siloxane-Ti material to form an apatite-like mineral is likely associated with the specific crystalline phase of the titania, as observed in the XRD patterns (Figure 4A,C). In addition, the SBF is a highly concentrated solution of sodium and chloride ions that becomes more concentrated as the aqueous medium evaporates, along with the presence of hydrophobic functional groups that restrict interfacial interactions, causing halite precipitation on the Siloxane-Ti surface, as seen in XRD (Figure 4C) and SEM (Figure 6) analyses. The literature suggests that while the anatase phase of titania is highly effective at inducing apatite precipitation, amorphous titania gels are ineffective, and the presence of rutile can further diminish this ability [14,46]. Nevertheless, Wu et al. [47] attributed the apatite-forming ability of anatase/rutile layers, after immersion in SBF, to both the epitaxial effect and the formation of Ti-OH groups during heat treatment at 60 or 80 °C with H_2_O_2_.

Apatite-forming ability serves as a material bioactivity indicator and demonstrates potential for application as an agent to regenerate bone tissue. In the present work, the Siloxane-Ti-Ca hybrid, after interacting with PBS and SBF media, showed the ability to form calcium-phosphate/apatite-like deposits. The lack of Ca ions and the presence of a rutile phase prevent this material from forming such deposits in both media.

### 3.5. Osteoblast-like Cell Adhesion and Morphology

To determine whether the proposed materials are suitable candidates for bone regeneration, the biocompatibility and bioactivity of the siloxane-Ti and siloxane-Ti-Ca hybrid materials, as well as changes in cell morphology and cell proliferation of MG-63 osteoblast-like cells, were analyzed.

We examined the organization of the actin cytoskeleton as an indicator of cell adhesion and spreading. We observed that osteoblasts adhered predominantly to siloxane-Ti hybrid materials, maintaining their characteristic spindle-shaped morphology with longitudinally arranged actin filaments. Staining revealed consistent cytoskeletal organization at all examined time points, with more pronounced filament distribution on days 14 and 21. SEM analysis further confirmed progressive cell proliferation on the surface of the Siloxane-Ti material: few cells were present on day 7, but on day 14 cell density increased, and on day 21 a near-confluent monolayer was observed.

Siloxane-Ti-Ca also supported cell adhesion; however, it showed lower cell density and reduced confluence at later culture stages than Siloxane-Ti (Figure 7). These results indicate that Ti-containing hybrids provide a more favorable surface for osteoblast adhesion, growth, and cytoskeletal organization. The strong adhesion is likely related to the chemical nature of TiO_2_-modified surfaces, where surface hydroxyl (Ti–OH) groups are known to mediate bioactivity. Several mechanisms can contribute to these interactions, including protein adsorption from the culture medium, protein secretion by the cells themselves, and direct physicochemical interactions with the substrate [48].

SEM observations (Figure 8) provided additional insights into osteoblast ultrastructure on siloxane hybrid materials. Cells developed lamellipodia and filopodia that established contact with both the biomaterial surface and neighboring cells. These protrusions, present at all studied time points, facilitate adhesion to the substrate (white arrows) and intercellular communication (empty arrows), processes that collectively enhance cell proliferation and growth. In general, it is well established that osteoblast cell adhesion is the initial step in early osseointegration, responding to bone material implants [49]. The cells cultured on rougher surfaces tend to display features of more differentiated osteoblasts compared with smoother surfaces, including lower cell density but higher alkaline phosphatase activity, a marker of early osteogenic differentiation [50].

Moreover, osteoblast adhesion on biomaterials also causes subsequent effects on activating intracellular signaling pathways that can regulate cell spreading, proliferation, and differentiation [49]. These results are aligned with a systematic review of titanium surfaces, which confirmed that microstructural and chemical cues strongly influence proliferation, differentiation, and protein synthesis of MG-63 cells [51].

### 3.6. Cell Viability, Proliferation, and Osteogenic Protein Expression Profiles

To detect potential adverse effects of the Siloxane-Ti and Siloxane-Ti-Ca on cell viability, we assessed cell cytotoxicity at 7, 14, and 21 days (Figure 9). Live/dead cell staining revealed predominantly green nuclei, confirming cell viability and indicating that no composition exhibited acute cytotoxicity at 7 and 14 days. However, after 21 days, Siloxane-Ti-Ca material showed a more pronounced reduction in viability (Figure 9A). Semi-quantitative analysis (100 cells per field) corroborated these observations (Figure 9B). Viability on Siloxane-Ti remained above 75% at all time points, peaking at >90% on day 14. In contrast, Siloxane-Ti-Ca showed a sharp decline on day 21, with cell viability dropping to ~10%. Furthermore, MTT assays were performed to complement cell viability and proliferation testing (Figure 9C). Siloxane-Ti material supported continuous cell growth over 21 days, while Siloxane-Ti-Ca also showed progressive growth but at consistently lower levels. These results indicate that while both hybrids are biocompatible, Siloxane-Ti provides a more favorable surface for sustained osteoblast proliferation.

The contrast in the findings is noteworthy, given that Ca-based biomaterials have been associated with strong biocompatibility due to their capacity to simulate the chemical composition and microstructure of natural bone tissues [52]. Moreover, the incorporation of calcium in biomaterials optimizes porosity, surface architecture, and resorption rates to increase the bone regeneration efficiency [53]. In the hybrids synthesized in this work, the presence of calcium changed their topography drastically (Figure 2C), being optimal only for adhesion and proliferation of the osteoblast-like cells during the first 14 days; from day 21, there was a severe decrease in viability of osteoblast-like cells on this material. This effect may be due to changes in the structure and/or surface properties of these hybrid materials, as has been reported in titanium implants with different structural and chemical surfaces [54]. According to the results mentioned previously, Siloxane-Ti-Ca hybrid materials showed a less interconnected siloxane matrix and a weight loss after 21 days of incubation in PBS and SBF. When this material was submerged in osteoblast culture medium (α-MEM supplemented with 10% FBS), it showed an eroded surface (Figure 9D), possibly associated with the release of high concentrations of Ca^2+^ ions that could cause variations in the cell–biomaterial microenvironment, limiting cell survival. It is known that Ca^2+^ regulates many cellular processes as a second messenger in cell signaling. However, high levels are related to reactive oxygen species production and mitochondrial damage, resulting in oxidative stress and apoptosis [55]. Also, the eroded surface results from hydrolysis of the highly hydrophilic siloxane matrix, which forms silicic acid in the culture medium. This process alters the hydrophilic/ hydrophobic ratio and modifies surface biocompatibility. On the other hand, since the experiments were conducted in a static system, it is possible that after 14 days, the cellular debris resulting from the first wave of cellular proliferation remained trapped in the interconnected particles and agglomerate domains, particularly present on the surface of the Siloxane-Ti-Ca hybrid material, reducing its biocompatibility. Although the same static system was used to analyze the Siloxane-Ti hybrid materials, their surface, with less roughness, possibly avoided the accumulation of cellular debris.

Furthermore, MTT assays were performed to complement cell viability and proliferation testing (Figure 9C). Siloxane-Ti material supported continuous cell growth over 21 days, while Siloxane-Ti-Ca also showed progressive growth but at consistently lower levels. These results indicate that while both hybrids are biocompatible, Siloxane-Ti provides a more favorable surface for sustained osteoblast proliferation.

To examine cell differentiation, the expression of osteoblast-related proteins was measured in the osteoblast-like cells grown on the hybrids. In the immunofluorescence analysis, we observed that osteonectin, an early marker in osteoblast maturation, was differentially expressed in the Siloxane-Ti at 1 and 21 days of culture (Figure 10A,D). On day 1, osteonectin expression was present only in a few cells, contrary to day 21, when the detection of this protein showed a homogeneous distribution in cells grown on this material. In contrast, in Siloxane-Ti-Ca hybrids, the expression of osteonectin was lacking in cells throughout all culture days (Figure 10A,D), which could be related to the absence of viable cells on these hybrids at 21 days. Our findings are consistent with reports describing osteonectin as a marker of early matrix organization in MG-63 cells [56,57]. Using a different proteomic technique, the Dot blot assay, we demonstrated that osteopontin expression began on day 7 in both hybrid materials, increasing over time and indicating that both compositions stimulated an intermediate stage of differentiation (Figure 10B). When we evaluated the expression of osteocalcin, a late marker of osteoblast maturation, we observed that it was detected from day 1 in Siloxane-Ti and only from day 7 in Siloxane-Ti-Ca (Figure 10C). However, osteocalcin production was similarly detected in supernatants of both hybrid materials at the next points analyzed. Taken together, our findings suggest that both hybrid materials promote the differential expression of proteins related to osteoblast maturation and differentiation. Nevertheless, the further use of unspecialized cells such as mesenchymal stem cells and the analysis of other proteins related to osteoblast differentiation, such as alkaline phosphatase, collagen I, and bone morphogenetic protein-2 [58], could confirm the osteoinductive capacity of siloxane hybrid materials.

## 4. Conclusions

In this work, we successfully synthesized hybrid materials based on siloxanes modified with TiO_2_ and/or Ca by the sol-gel method. We evaluated their physicochemical properties and compositional changes as well. Additionally, we assessed how these materials interact with osteoblast-like cells and the response of this cell line to them. The composition of the hybrids strongly influenced both their stability and bioactivity. Siloxane-Ti exhibited a dense, rutile-containing network with excellent stability in PBS, no apatite induction, and favorable biological behavior, supporting adhesion, proliferation, and differentiation of osteoblasts. In contrast, Siloxane-Ti-Ca presented a more open and porous structure, released Ca^2+^, and induced rapid flake/cauliflower apatite precipitation. However, despite the apatite formation, the Siloxane-Ti-Ca hybrid presented limited viability and cell growth at 21 days, in addition to reduced expression of markers of osteoblast differentiation, which limits its possible biomedical application but opens the door to studying other factors that can influence biocompatibility and osteoinductive and osteoconductive capacity of this hybrid over long periods of time.

These findings emphasize the need to balance mineralization capacity and cell compatibility when designing Siloxane hybrids. While TiO_2_ incorporation alone provides stable and cell-supportive surfaces, the addition of Ca^2+^ introduces bioactivity but at the expense of cell proliferation and differentiation. Further optimization of composition and processing will be necessary to fine-tune the degradation rate, surface chemistry, and bioactivity of these materials.

## Figures and Tables

**Figure 1:**
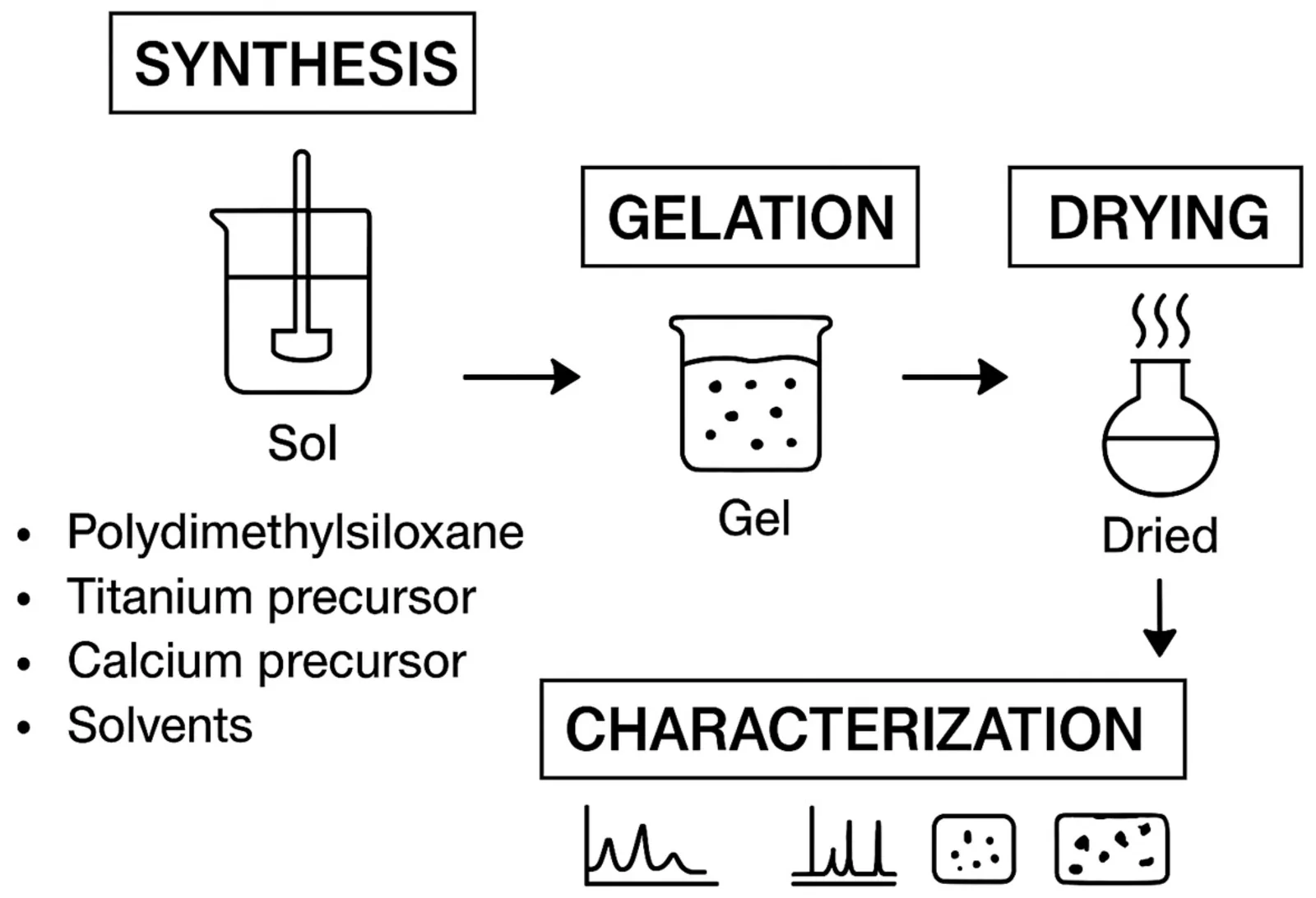
Graphical workflow from hybrid materials synthesis to characterization.

**Figure 2:**
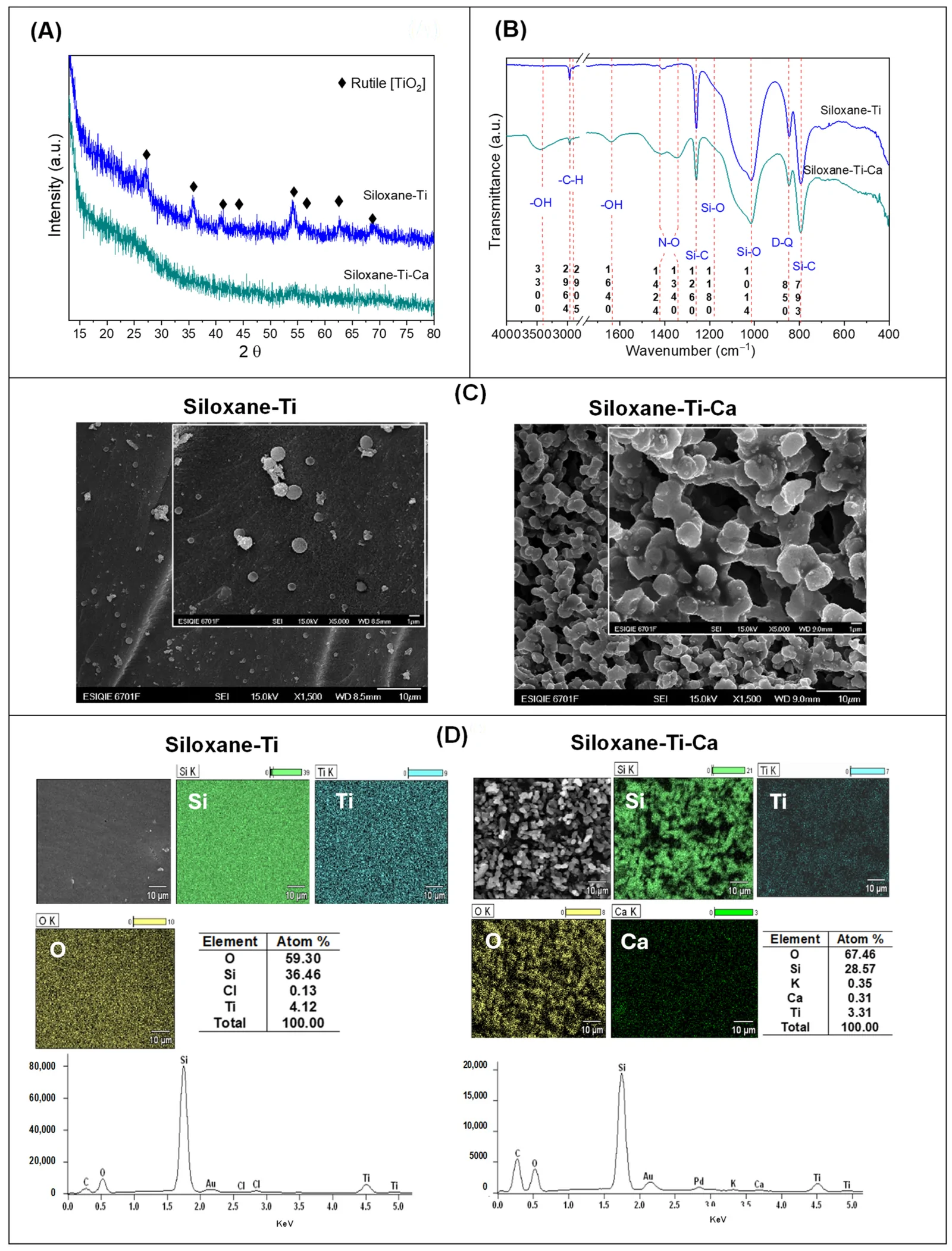
(**A**) XRD, (**B**) FT-IR, (**C**) SEM analysis, and (**D**) EDS microanalysis of Siloxane-Ti and Siloxane-Ti-Ca hybrids.

**Figure 3:**
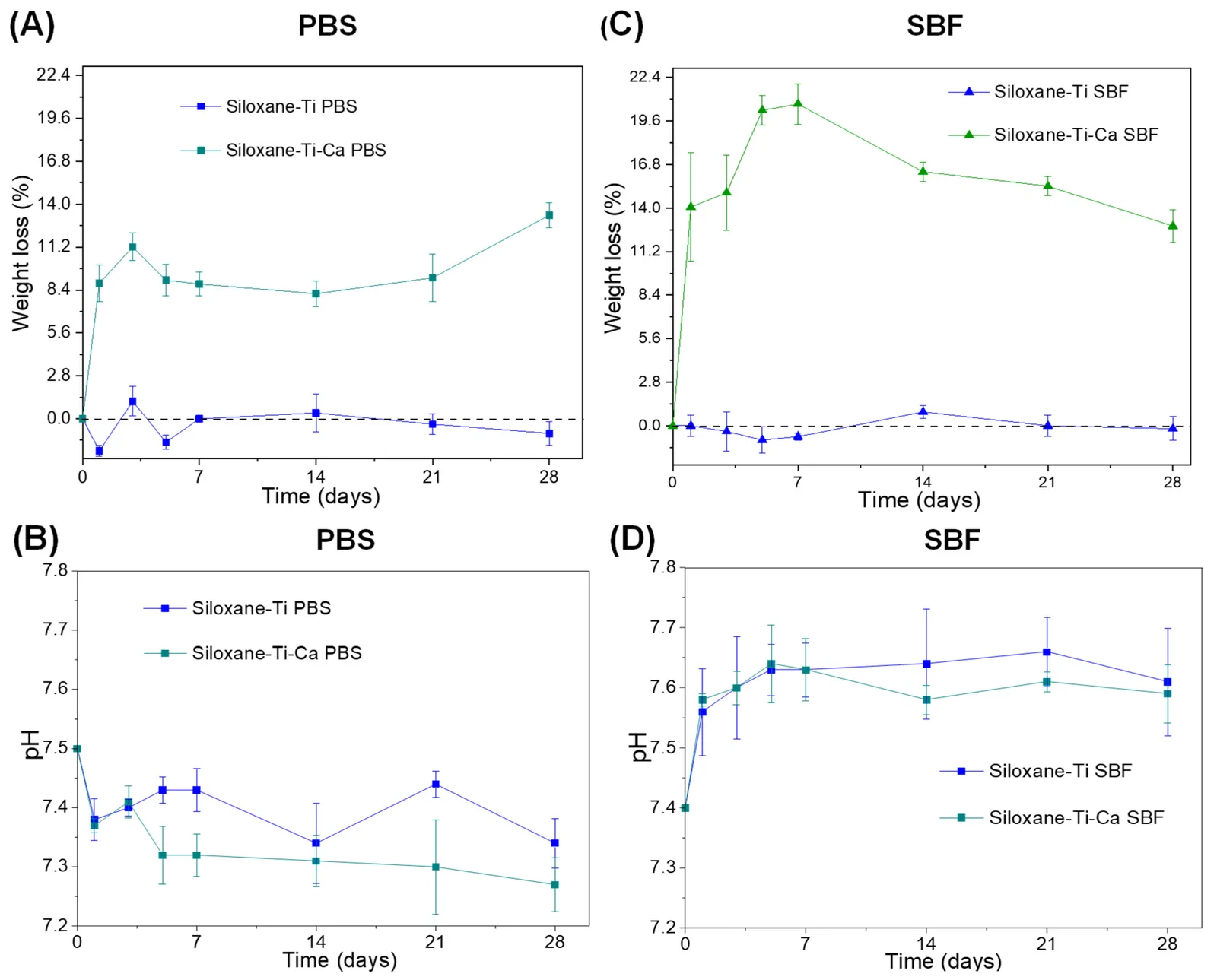
(**A**) Weight and (**B**) pH variation of Siloxane-Ti and Siloxane-Ti-Ca hybrid materials in PBS; (**C**) Weight and (**D**) pH variation of Siloxane-Ti and Siloxane-Ti-Ca hybrid materials in SBF during testing time.

**Figure 4:**
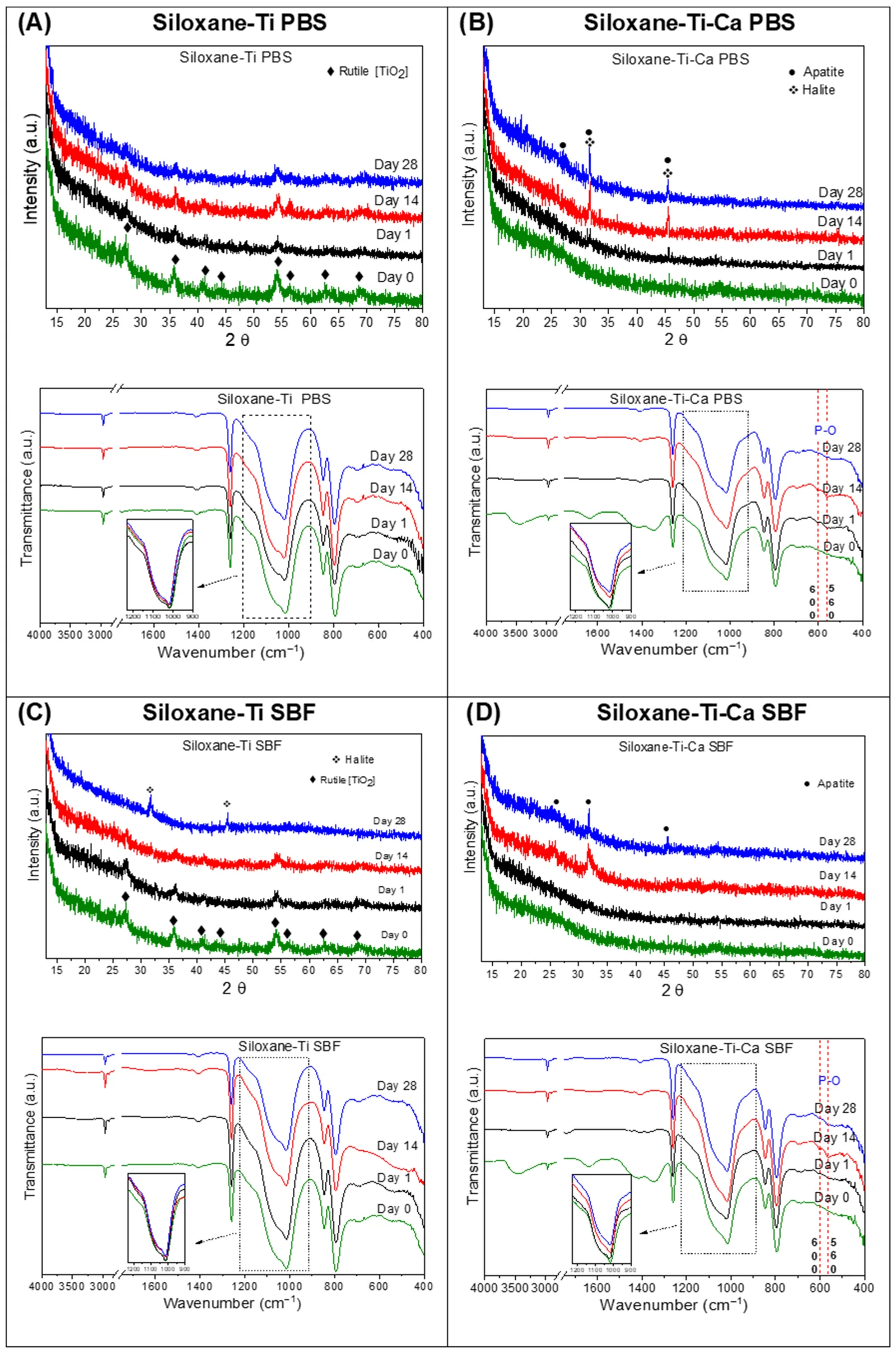
XRD and FT-IR of (**A**) Siloxane-Ti and (**B**) Siloxane-Ti-Ca hybrid materials after immersion in PBS; (**C**) Siloxane-Ti and (**D**) Siloxane-Ti-Ca hybrids after immersion in SBF during the testing time.

**Figure 5:**
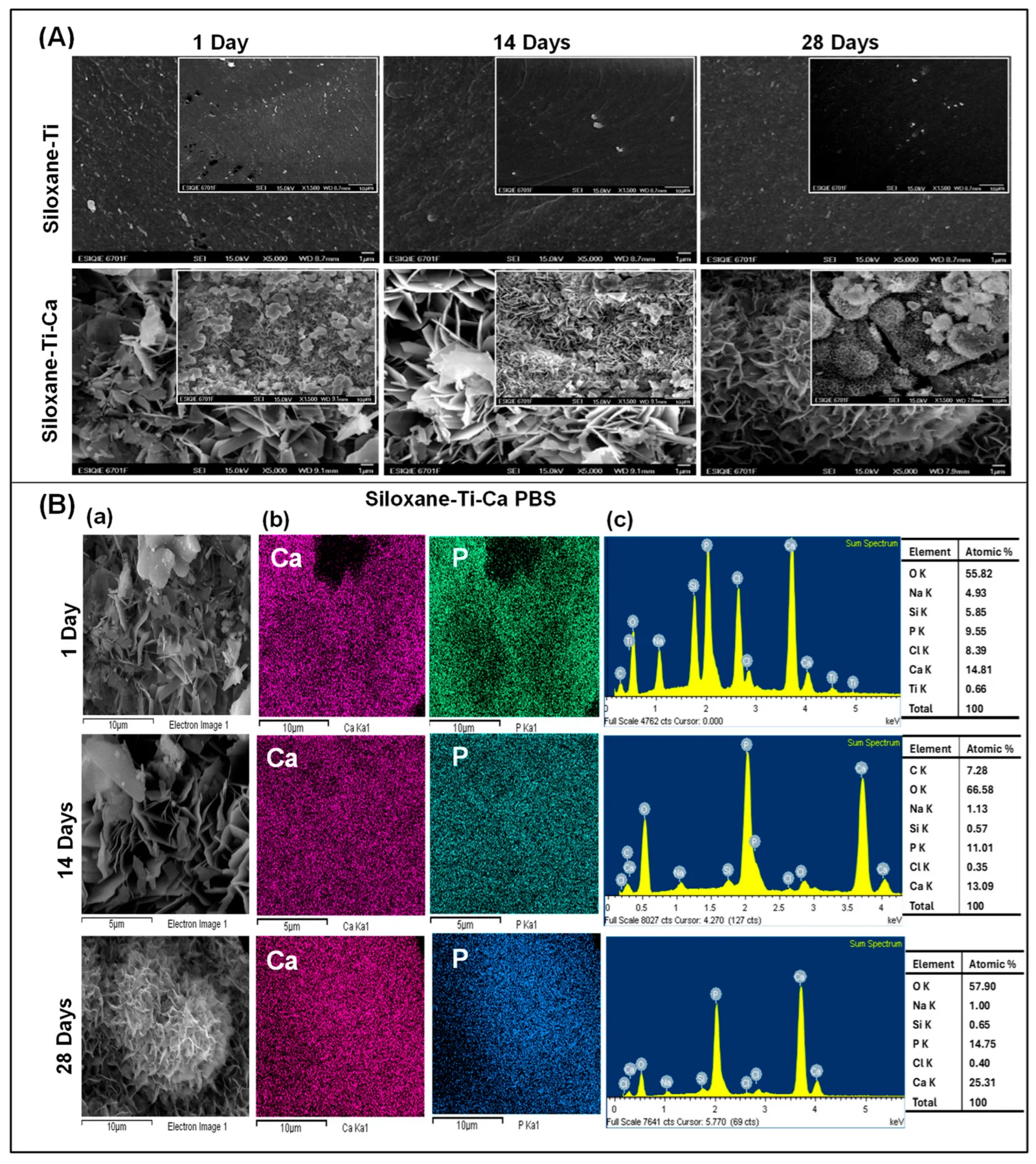
(**A**) SEM images of Siloxane-Ti and Siloxane-Ti-Ca hybrid materials after immersion in PBS during the testing period. (**B**) SEM-EDS analysis of Siloxane-Ti-Ca after immersion in PBS during the testing period: (**a**) SEM micrograph and corresponding EDS elemental mapping. (**b**) The mapping highlights the distribution of Ca and P elements. (**c**) EDS spectra and quantitative elemental values.

**Figure 6:**
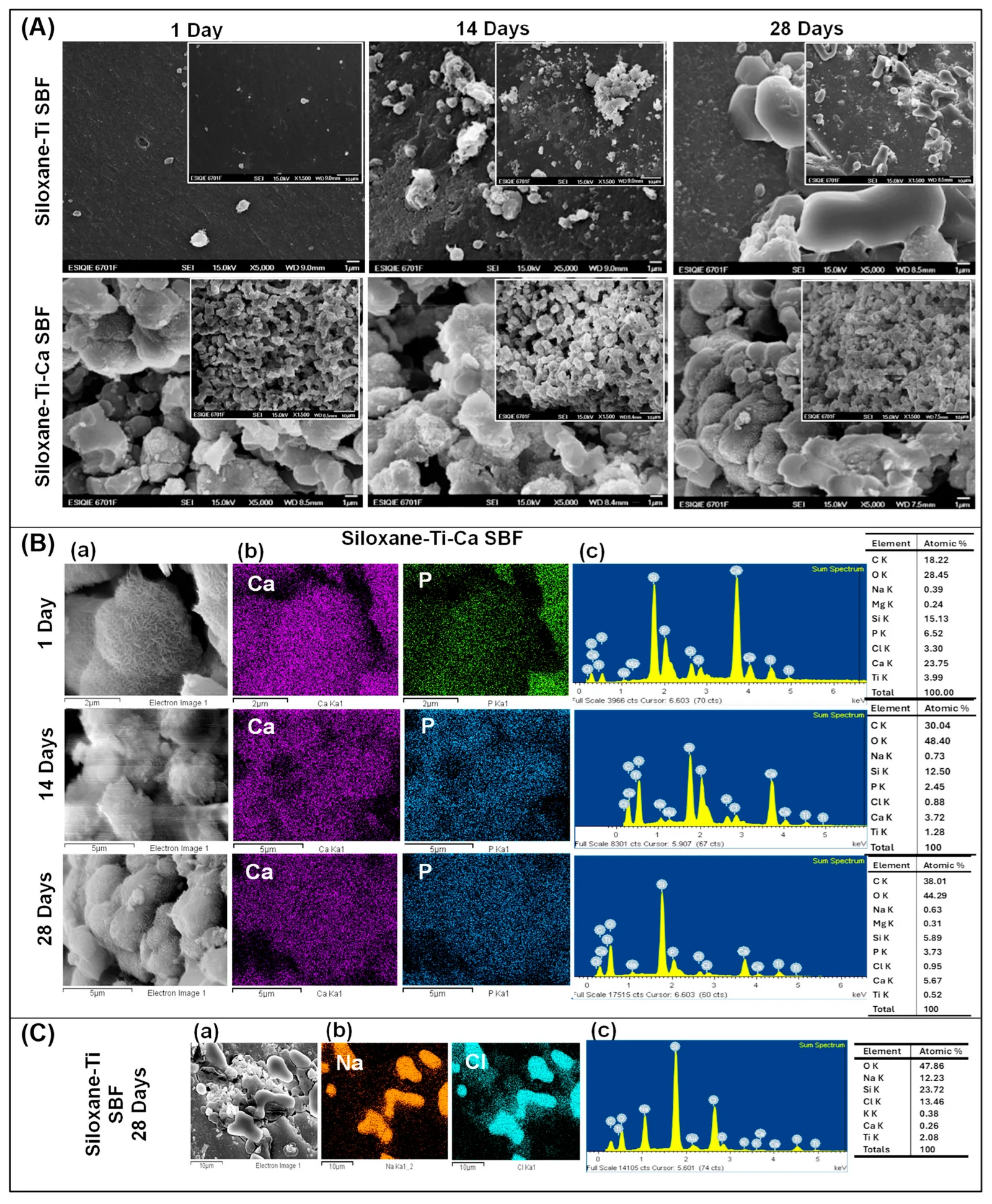
(**A**) SEM images of Siloxane-Ti and Siloxane-Ti-Ca hybrid materials after immersion in SBF. (**B**) SEM-EDS analysis of Siloxane-Ti-Ca after immersion in SBF during the testing period: (**a**) SEM micrograph and corresponding EDS elemental mapping. (**b**) The mapping highlights the distribution of Ca and P elements. (**c**) EDS spectra and quantitative elemental values. (**C**) SEM-EDS analysis of Siloxane-Ti after 28 days of immersion in SBF: (**a**) SEM micrograph and corresponding EDS elemental mapping. (**b**) The mapping highlights the distribution of Na and Cl elements. (**c**) EDS spectrum and quantitative elemental values.

**Figure 7:**
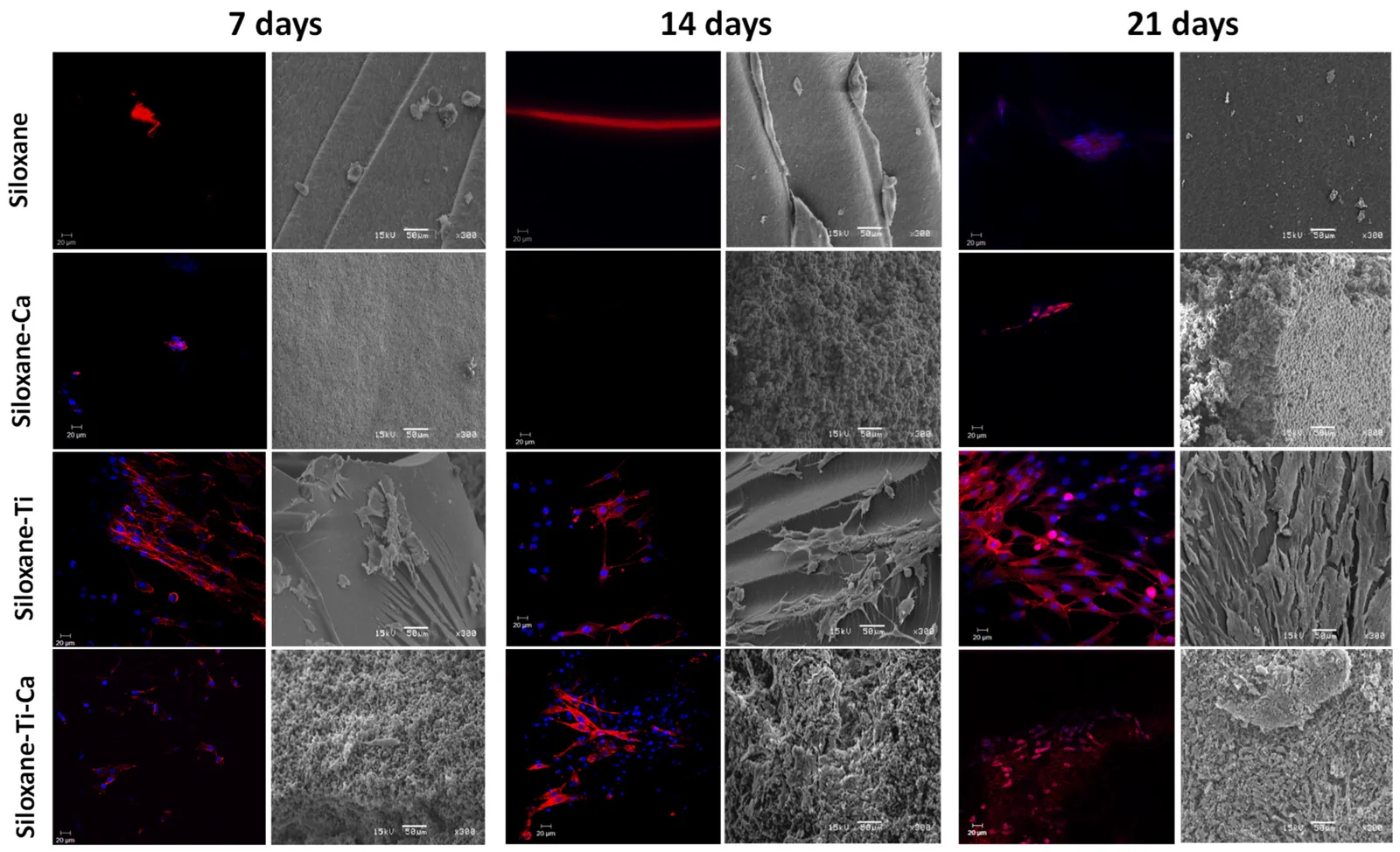
Cell adhesion and cytoskeletal arrangement of osteoblast-like cells grown on siloxane-hybrid materials. Fluorescence images show the actin cytoskeleton (red) stained with phalloidin-TRITC, and the cell nucleus (blue) stained with DAPI. Scanning electron microscopy images show the cells attached to the surface of the materials. The images are representative of two independent experiments. Scale bars indicate 20 µm in fluorescence images and 50 µm in SEM images.

**Figure 8:**
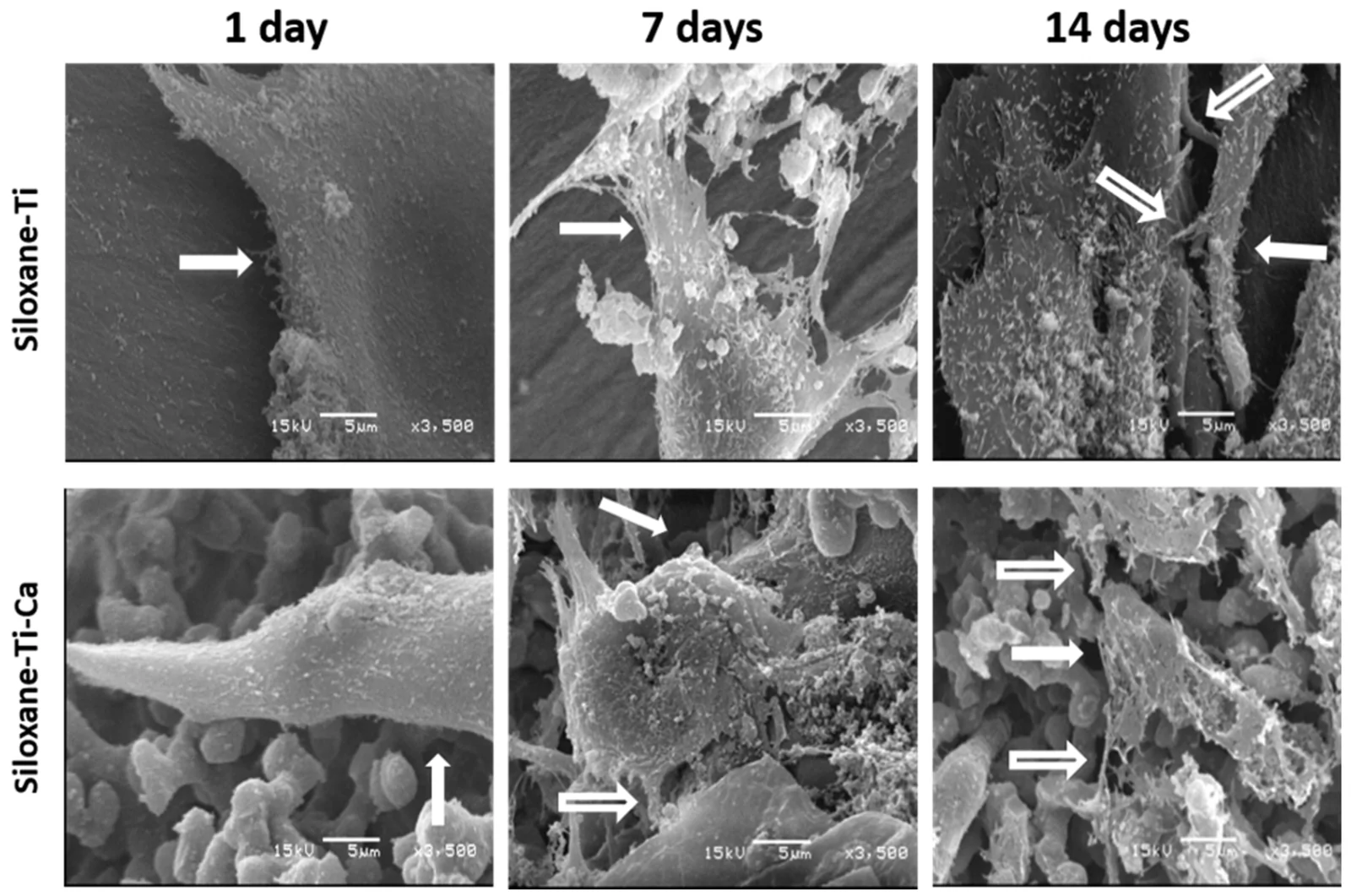
MG-63 osteoblast-like cells ultrastructure cultured on siloxane hybrid materials over time. The interaction of cell-to-cell extensions is shown with empty arrows. The interaction of cell-to-biomaterials is indicated with filled arrows. Scale bars indicate 5 µm in all images.

**Figure 9:**
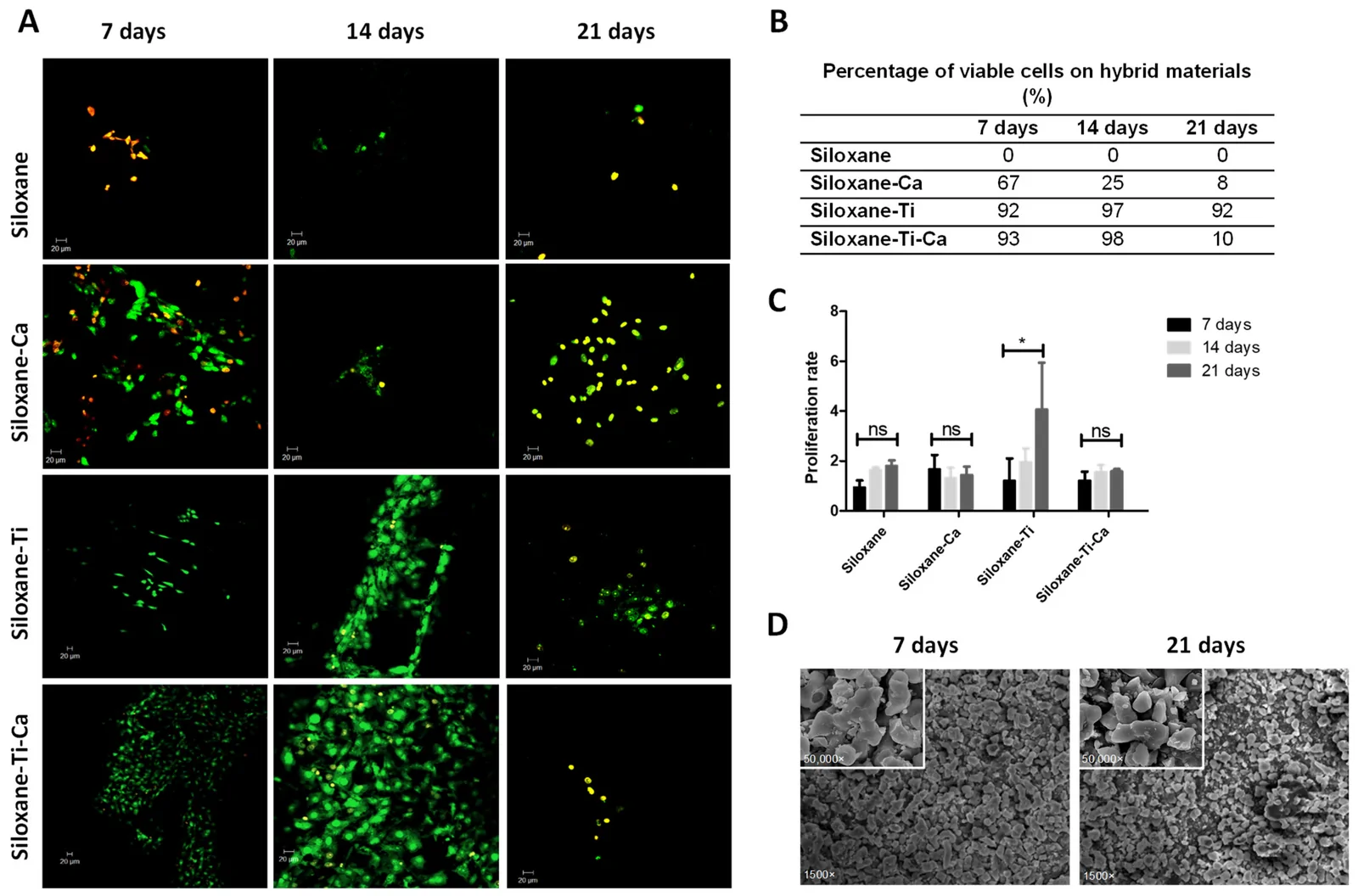
Cell viability and proliferation rate of osteoblast-like cells cultured on Siloxane hybrids. (**A**) Cell viability at 7, 14, and 21 days; a mixture of SYTO9 and propidium iodide was used to distinguish living cells (green) and dead cells (red). Images are representative of two independent experiments. (**B**) Semi-quantitative analysis of viable cells on hybrid materials. The percentage of viable cells was obtained from confocal images; 100 cells per field were quantified to calculate this percentage. (**C**) Proliferation rate at 7, 14, and 21 days. Cells were cultured on the hybrid material surface, and proliferation was evaluated by MTT assay. The proliferation rate was calculated, taking the optical density of day 1 as representative of the cells initially adhered to the hybrids. Bars represent the mean ± SD of three independent experiments in triplicate. The data were analyzed using GraphPad Prism software, version 4.03. Statistical differences between experimental groups and conditions were evaluated using two-way analysis of variance (ANOVA) followed by Tukey’s post hoc test. A *p*-value of <0.05 * was considered statistically significant; ns = not significant. (**D**) SEM images representative of the Siloxane-Ti-Ca hybrids submerged in α-MEM supplemented with 10% FBS at 7 and 21 days. Magnification 1500x. The small squares show 50,000× magnification.

**Figure 10:**
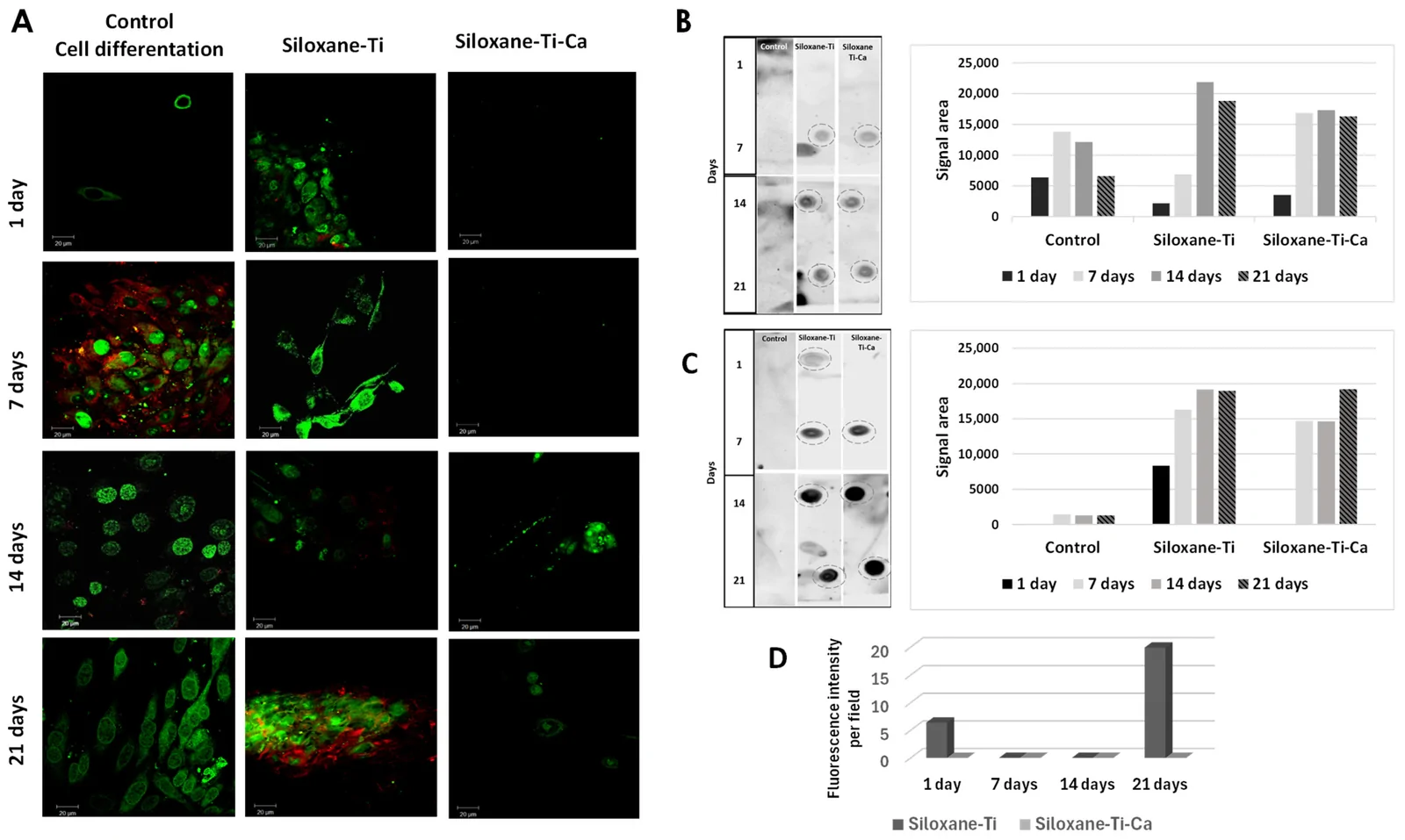
Cell differentiation of osteoblast-like cells cultured on the siloxane hybrid materials surface. (**A**) Osteonectin expression at 1, 7, 14, and 21 days; osteonectin (red) and nuclei (green) were detected by immunofluorescence. Control cell differentiation represents cells stimulated by β-glycerophosphate as an osteogenic control. Images are representative of two experiments. Scale bars represent 20 µm. Osteopontin (**B**) and osteocalcin (**C**) were detected by Dot blot. Membranes are representative of a triplicate experiment. Dashed circles in membranes indicate the resulting dots after reveal. Graphs represent the semi-quantitative analysis of pixel intensity of the dots present in the nitrocellulose membranes. (**D**) Semi-quantitative analysis of fluorescence intensity per field of osteonectin expression is shown in (**A**).

**Table 1:** Composition of siloxane hybrid samples.

Sample ID	Inorganic Components	Organic Component	Calcium Source	Inorganic/Organic Mass Ratio	Calcium Content (wt%)	Final Notes
Siloxane-Ti	TEOS + Ti(OiPr)_4_	PDMS	–	(0.45 + 0.20)/0.35	–	Rutile traces, dense morphology.
Siloxane-Ti-Ca	TEOS + Ti(OiPr)_4_	PDMS	Ca(NO_3_)_2_·4H_2_O	(0.45 + 0.20)/0.35	~1 wt%	Porous surface, enhanced apatite precipitation

## Data Availability

The raw data supporting the conclusions of this article will be made available by the authors on request.
